# Occupational depression among beverage-processing workers in East Java, Indonesia: a cross-sectional study of work-related stressors

**DOI:** 10.3389/fpubh.2026.1771289

**Published:** 2026-03-24

**Authors:** Yusrin Aulia, Agustina Konginan, Atika, Aji Muthiah Nur Azizah, Rubayat Indradi

**Affiliations:** 1 Faculty of Medicine, Department of Psychiatry, Dr. Soetomo General Hospital, Universitas Airlangga, Surabaya, Indonesia; 2 Fakultas Kedokteran, Universitas Muhammadiyah Malang, Malang, Indonesia

**Keywords:** factory workers, mental health, occupational depression, quantitative workload, work stress

## Abstract

**Background:**

Depression is a frequent mental health problem in industrial workers. Understanding work-related stressors linked to occupational depression can guide preventive action in industry settings. Objective: To analyze associations between work stress factors and occupational depression among production workers in a beverage processing factory in East Java, Indonesia.

**Methods:**

An observational analytic cross-sectional mixed-methods (explanatory sequential) study was conducted between October 2024 and April 2025. Quantitative data were collected using self-administered questionnaires covering demographics, the Holmes–Rahe Social Readjustment Rating Scale, the Stress Diagnostic Survey-30 (SDS-30), and the Occupational Depression Inventory (ODI). Fifteen participants were randomly selected for focus group discussions (FGDs) to explore perceived workplace stressors. Occupational depression was defined as an ODI score ≥5. Associations were examined using a chi-squared or Fisher’s exact test and multivariable logistic regression analysis.

**Results:**

Of the 140 production workers, 28 (20.0%) screened positive for occupational depression. In bivariate analyses, all six SDS-30 domains—role ambiguity, role conflict, quantitative overload, qualitative overload, career development stress, and responsibility for others—were significantly associated with occupational depression (*p* < 0.05). In multivariable analysis, moderate-to-severe quantitative overload remained independently associated with occupational depression (adjusted OR 5.18; 95% CI 2.06–13.06).

**Discussion:**

Occupational depression affected one in five production workers in this setting. Quantitative workload pressure emerged as the most salient and modifiable risk factor, underscoring the importance of workload management, clear task allocation, and organizational support to protect workers’ mental health.

## 1 Introduction

Depression, a prevalent mental disorder marked by persistent low mood, anhedonia, cognitive impairment, and functional decline, affects an estimated 280 million individuals worldwide—approximately 3.8% of the global population (1). In Indonesia, the prevalence is comparable, with recent estimates indicating that 3.7% of the national population (≈ 3.9%) experiences depressive disorders (2). However, data on occupational depression within the Indonesian workforce remain scarce (3).

Industrial workers are disproportionately vulnerable owing to demanding production environments, shift work, and psychosocial stressors (4). Empirical evidence suggests that the prevalence of depressive symptoms among industrial employees may exceed that of the general population (2, 3). A comprehensive meta-review spanning three decades of research further demonstrates consistent associations between psychosocial job stressors—such as workload pressure, role ambiguity, and job insecurity—and adverse mental health outcomes, including depression (21). Reported rates in industrial cohorts reach as high as 21%, surpassing the 18–20% observed in Europe and North America (5). In Indonesia, 60.6% of workers in small- and medium-sized enterprises reported depressive symptoms in 2017 (6), underscoring the impact of unhealthy work environments, poor interpersonal dynamics, and chronic exposure to unresolved job problems on mental health (1, 16).

The Beverage Processing Plant in East Java—one of Indonesia’s largest facilities within a global production network—employs over 1,000 workers and adheres to stringent international operating standards. The plant integrates advanced technology and comprehensive health-and-safety programmes, including annual occupational health examinations that routinely assess work-related stress. Although these examinations have identified a proportion of employees experiencing moderate to severe stress, no systematic evaluation has linked such stress to occupational depression.

Given the heightened risk of depression among industrial workers and the paucity of data linking work-related stress to depressive outcomes in this setting, the present study aims to (i) examine associations between key psychosocial work-stress dimensions—including role ambiguity, role conflict, quantitative overload, qualitative overload, career-development stress, and responsibility for others—and occupational depression among beverage-processing workers; (ii) develop a predictive scoring system to identify individuals at risk; and (iii) inform preventive policies that target modifiable stressors. By elucidating workplace risk profiles, this investigation seeks to foster targeted interventions, improve mental health outcomes, and sustain productivity in a demographically diverse workforce.

## 2 Methods

### 2.1 Study design

This study employed an observational analytic cross-sectional design with an explanatory sequential mixed-methods approach, in which quantitative data collection was followed by qualitative exploration through focus group discussions (FGDs) to provide contextual interpretation of the quantitative findings (Table 1).

**Table 1:** Bivariate associations.

Factor	Category	Not depressed (*n* = 112)	Depressed (*n* = 28)	Total (*n* = 140)	*p*
Sex	Male	110 (98.2)	25 (89.3)	135	0.055a
	Female	2 (1.8)	3 (10.7)	5	
Age	18–40	67 (59.8)	18 (64.3)	85	0.665
	41–55	45 (40.2)	10 (35.7)	55	
Education	< High school	3 (2.7)	1 (3.8)	4	1.000a
	≥ High school	109 (97.3)	27 (96.4)	136	
Marital	Married	112 (100)	27 (96.4)	139	0.200a
	Not married	0	1 (3.6)	1	
Role ambiguity	Light	69 (61.6)	11 (39.3)	80	0.033*
	Mod+Sev	43 (38.4)	17 (60.7)	60	
Role conflict	Light	66 (58.9)	9 (32.1)	75	0.011*
	Mod+Sev	46 (41.1)	19 (67.9)	65	
Quantitative overload	Light	76 (67.9)	8 (28.6)	84	<0.001*
	Mod+Sev	36 (32.1)	20 (71.4)	56	
Qualitative overload	Light	65 (58.0)	8 (28.6)	73	0.005*
	Mod+Sev	47 (42.0)	20 (71.4)	67	
Career development	Light	58 (51.8)	8 (28.6)	66	0.028*
	Mod+Sev	54 (48.2)	20 (71.4)	74	
Responsibility others	Light	67 (59.8)	7 (25.0)	74	0.001*
	Mod+Sev	45 (40.2)	21 (75.0)	66	

A Fisher’s exact test where appropriate. *p < 0.05.

### 2.2 Study setting and period

The study was conducted at a beverage processing factory in East Java, Indonesia. Data collection took place from October 2024 to April 2025. As the study was limited to a single workplace setting, the findings may not be fully generalizable to other industrial contexts (Table 2).

**Table 2:** Multivariable logistic regression.

Factor	Category	*B*	*p*	OR	95% CI
Sex	Female vs. Male	1.802	0.076	6.059	0.827–44.398
Quantitative overload	Mod+Sev vs. Light	1.645	<0.001	5.181	2.055–13.059

Model Nagelkerke R Square = 18.6%.

### 2.3 Study population

The target population consisted of production-area employees aged 18–55 years, with a minimum of 5 years of continuous service and working under a three-shift rotation system.

Employees were excluded if they had a previous clinical diagnosis of depression or a history of antidepressant use prior to employment, or if they demonstrated high non-occupational life stress, defined as a Holmes–Rahe Social Readjustment Rating Scale score ≥300, to minimize confounding effects unrelated to workplace stressors.

### 2.4 Sample size and sampling

The minimum sample size was calculated using a two-proportion comparison formula, yielding a required sample of 138 participants. A total of 140 workers meeting the eligibility criteria were recruited for the quantitative survey.

Purposive sampling was applied to ensure inclusion of eligible workers across production units. For the qualitative component, 15 participants were selected using simple random sampling stratified by work area and assigned to three FGDs.

### 2.5 Data collection instruments

- Data were collected using the following instruments:
- A demographic questionnaire capturing age, sex, education level, marital status, and work tenure.
- The Holmes–Rahe Social Readjustment Rating Scale (Indonesian-adapted version) to screen for high levels of non-work-related life stress.
- The Stress Diagnostic Survey-30 (SDS-30), which assesses six psychosocial workplace stress domains: role ambiguity, role conflict, quantitative overload, qualitative overload, career development, and responsibility for others.
- The Occupational Depression Inventory (ODI), which assesses depressive symptoms explicitly attributed to work conditions. An ODI score ≥5 was used to classify participants as experiencing occupational depression, in accordance with established guidelines.
- Severity levels of SDS-30 domains were categorized based on validated scoring cut-off points. The observed concentration of scores within the moderate to severe range reflects the distribution of responses near category thresholds rather than limitations in data handling or sample size.

### 2.6 Data collection procedures

Quantitative data were collected using self-administered questionnaires distributed via online Google Forms during routine medical check-ups, with paper-based surveys provided during shift breaks to ensure inclusivity and maximize participation. Quantitative data were collected using self-administered questionnaires distributed via online Google Forms during routine medical check-ups, with paper-based surveys provided during work breaks to ensure inclusivity and maximize participation.

FGDs were conducted in three groups, each lasting approximately 30 min, and focused on participants’ perceptions of workplace stressors, job demands, and mental health experiences. FGDs were conducted in three groups, each lasting approximately 30 minutes, and focused on participants’ perceptions of workplace stress, job demands, and mental health experiences.

### 2.7 Study variables

The primary outcome variable was occupational depression, defined as an ODI score ≥5.

Independent variables included demographic characteristics (age group, sex, education level, marital status, and work tenure) and the six SDS-30 psychosocial stress domains, which were dichotomized into light vs. moderate-to-severe categories.

### 2.8 Statistical analysis

Descriptive statistics were used to summarize participant characteristics and variable distributions.

Bivariate associations between independent variables and occupational depression were analyzed using the chi-squared test or Fisher’s exact test when expected cell counts were less than five. Variables with *p*-values <0.25 in bivariate analyses were entered into a multivariable logistic regression model using a backward elimination approach to estimate adjusted odds ratios (ORs) with 95% confidence intervals (CIs). Statistical significance was set at *p* < 0.05.

Although sex was recorded as a demographic variable, sex-stratified analyses were not emphasized due to a substantial imbalance in group sizes, which could compromise statistical validity. Therefore, sex was treated primarily as a descriptive characteristic in the analysis.

### 2.9 Ethical consideration

The study protocol was approved by the Institutional Ethics Committee (ethical clearance certificate No.: 157/EC/KEPK/FKUA/2024).

All participants provided written informed consent prior to participation. To ensure confidentiality and minimize response bias, data were collected anonymously without personal identifiers, and individual responses were not accessible to workplace managers or supervisors. Participants were informed that their responses would be used solely for research purposes and would not affect their employment status or performance evaluations. All data were analyzed in aggregate form.

## 3 Results

### 3.1 Participant characteristics

A total of 140 eligible production workers participated in the study (response rate: 100% following supplemental paper-based distribution). The majority of participants were male (96.4%), aged 18–40 years (60.7%), had at least a high school education (97.1%), and were married (99.3%).

The majority of respondents reported working primarily to support family income, earning <10 million IDR per month, commuting >10 km between home and workplace, and being members of a labor union.

Based on SDS-30 scoring, most psychosocial stress domains were distributed within the light to moderate range, with relatively low proportions of severe stress across domains. Overall, 20% of participants screened positive for occupational depression (ODI ≥ 5).

### 3.2 Bivariate associations

The prevalence of occupational depression differed significantly across all six SDS-30 psychosocial stress domains. Workers reporting moderate-to-severe stress had consistently higher rates of occupational depression compared with those reporting light stress, including role ambiguity (*p* = 0.033), role conflict (*p* = 0.011), quantitative overload (*p* < 0.001), qualitative overload (*p* = 0.005), career development stress (*p* = 0.028), and responsibility for others (*p* = 0.001).

No statistically significant associations were observed for sex (*p* = 0.055), age group (*p* = 0.665), education level (*p* = 1.000), or marital status (*p* = 0.200). Given the substantial imbalance in sex distribution, comparisons involving sex should be interpreted with caution.

### 3.3 Multivariable logistic regression

Variables with *p* < 0.25 in bivariate analyses were included in the multivariable logistic regression model. In the final model, moderate-to-severe quantitative overload remained independently associated with occupational depression (OR 5.18; 95% CI 2.06–13.06; *p* < 0.001).

Sex showed a non-significant trend, with a wide confidence interval (OR 6.06; 95% CI 0.83–44.40; *p* = 0.076), reflecting limited statistical precision due to the small number of female participants. The model explained 18.6% of the variance in occupational depression (Nagelkerke R^2^ = 0.186).

### 3.4 Qualitative findings (FGDs)

Three FGDs involving 15 male participants identified themes that supported and contextualized the quantitative findings. Participants described high quantitative and qualitative workload demands, frequent task carryover between shifts, and role expansion beyond prior training, contributing to fatigue and frustration.

Additional themes included uncertainty regarding promotion timelines, perceived inequities in career advancement decisions, and increased responsibility for team outcomes, particularly when tasks were left unfinished by previous shifts. These experiences were commonly linked to reduced motivation and emotional strain, reinforcing the role of workload-related stressors in occupational mental health.

## 4 Discussion

In this study, one in five production workers met the ODI threshold for occupational depression, a prevalence comparable to estimates reported in other industrial populations (5). Variability across studies is partly attributable to differences in screening instruments, as lower prevalence rates are often reported when using general depression measures such as the CES-D or PHQ-9 (7, 8). By explicitly attributing symptoms to work conditions, the Occupational Depression Inventory may offer greater sensitivity in detecting work-related psychological distress (9).

Several psychosocial domains—including role ambiguity, role conflict, quantitative overload, qualitative overload, career development stress, and responsibility for others—showed significant unadjusted associations with occupational depression. These findings are consistent with prior international research linking psychosocial work stressors to depressive outcomes (10–12). However, after multivariable adjustment, only quantitative overload remained independently associated, highlighting workload volume and time pressure as the most salient and potentially modifiable risk factors in this industrial setting (13, 17–20).

Qualitative findings further supported this interpretation, with workers describing high production targets, task carryover between shifts, unclear promotion timelines, and expanded responsibilities beyond prior training, all of which contributed to perceived overload and emotional strain.

A non-significant trend toward higher odds of occupational depression among female workers was observed. However, this finding should be interpreted cautiously due to the substantial imbalance in sex distribution and the resulting limited statistical precision, as reflected by the wide confidence interval. Future studies with more gender-balanced samples are needed to better examine potential sex-related differences in occupational mental health risk.

From a prevention perspective, these findings suggest that organizational-level interventions should prioritize managing quantitative workload, clarifying task expectations, and ensuring transparent career development processes. Strength-based career development approaches—emphasizing skill utilization, role clarity, and realistic advancement pathways—may further buffer the mental health impact of occupational stressors (22). Practical measures may include monitoring production targets, balancing shift-related task distribution, and training supervisors to recognize early signs of work-related psychological distress. Such strategies align with international organizational health recommendations (14, 15), as well as Indonesian occupational health regulations, emphasizing the management of psychosocial workplace hazards (6).

### 4.1 Limitations

This study has several limitations. The cross-sectional design precludes causal inference. Reliance on self-reported measures may introduce reporting bias, and the ODI serves as a screening tool rather than a clinical diagnostic instrument. Additionally, the study was conducted in a single industrial setting, which may limit generalizability to other sectors.

Importantly, the analysis did not account for certain contextually relevant socioeconomic and logistical factors, such as income adequacy, commuting distance, or employment-related migration, which are common in the Indonesian workforce and may influence occupational stress and mental health. Finally, the low representation of female workers limited the feasibility of sex-stratified analyses.

### 4.2 Implications for practice

Occupational health programs should integrate routine screening for work-related stressors and depressive symptoms, with particular attention to quantitative workload management. Developing equitable and transparent promotion systems, alongside supervisor training in workload allocation and early mental health referral, may help reduce occupational depression and improve workforce wellbeing (17).

## 5 Conclusion

Work-related stress—particularly quantitative workload pressure—is significantly associated with occupational depression among beverage factory workers in East Java. Addressing excessive workload demands and strengthening organizational support mechanisms may contribute to the prevention of work-related depression and the maintenance of sustainable productivity.

## Data Availability

The raw data supporting the conclusions of this article will be made available by the authors, without undue reservation.
