# Supplementary material for: Occupational depression among beverage-processing workers in East Java, Indonesia: a cross-sectional study of work-related stressors
Source: Front Public Health. 2026 Mar 24;14:1771289. doi: 10.3389/fpubh.2026.1771289 (PMC13053495; doi:10.3389/fpubh.2026.1771289)
Supplement: Supplementary file 1 [file Table_1.DOCX]

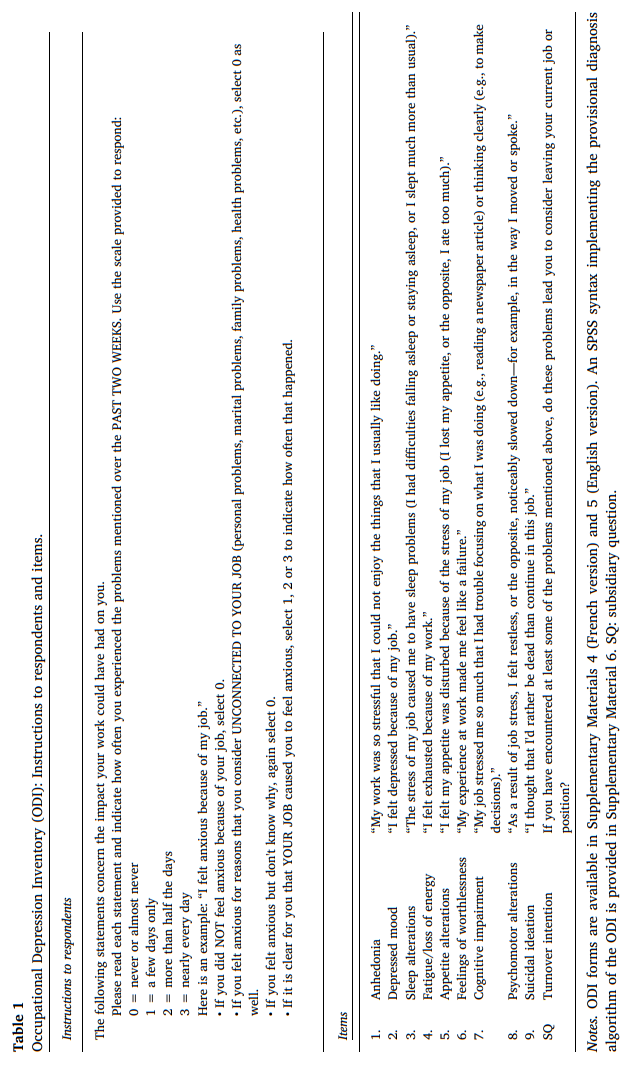


**Stress Diagnostic Survey-30 (SDS 30)**

The following questionnaire is designed to provide you with an indication of the extent to which various individual-level stressors are [sources of stress](https://scales.arabpsychology.com/glossary_tag/sources-of-stress/) to you. For each item you should indicate the frequency with which the condi­tion described is a source of stress. Next to each item write the appropriate number which best describes how frequently the condition described is a source of stress.

Write l if the condition described is never a source of stress.

Write 2 if it is rarely a source of stress.

Write 3 if it is occasionally a source of stress.

Write 4 if it is sometimes a source of stress.

Write 5 if it is often a source of stress.

Write 6 if it is usually a source of stress.

Write 7 if it is always a source of stress.

| **Condition** |  | | | | | | |
| --- | --- | --- | --- | --- | --- | --- | --- |
|  | **1** | **2** | **3** | **4** | **5** | **6** | **7** |
| 1. My job duties and work objectives are unclear to me. 2. I work on unnecessary tasks or projects. 3. I have to take work home in the evenings or on weekends to stay caught up. 4. The demands for work quality made upon me are unreasonable. 5. I lack the proper opportunities to advance in this organization. 6. I am held accountable for the development of other employees. 7. I am unclear about whom I report to and/or who reports to me. 8. I get caught in the middle between my supervisors and my subordinates. 9. I spend too much time in unimportant meetings that take me away from my work. 10. My assigned tasks are sometimes too difficult and/or complex. 11. If I want to get promoted, I have to look for a job with another organization. 12. I am responsible for counseling with my subordinates and/or helping them solve their problems. 13. I lack the authority to carry out my job responsibilities. 14. The formal chain of command is not adhered to. 15. I am responsible for an almost unmanageable number of projects or assignments at the same time. 16. Tasks seem to be getting more and more complex. 17. I am hurting my career progress by staying with this organization. 18. I take action or make decisions that affect the safety or well-being of others. 19. I do not fully understand what is expected of me. 20. I do things on the job that are accepted by one person and not by others. 21. I simply have more work to do than can be done in an ordinary day. 22. The organization expects more of me than my skills and/or abilities provide. 23. I have few opportunities to grow and learn new knowledge and skills in my job. 24. My responsibilities in this organization are more for people than for things. 25. I do not understand the part my job plays in meeting overall organizational objectives. 26. I receive conflicting requests from two or more people. 27. I feel that I just don’t have time to take an occasional break. 28. I have insufficient training and/or experience to discharge my duties properly. 29. I feel that I am at a standstill in my career. 30. I have responsibility for the future (careers) of others. |  |  |  |  |  |  |  |
